# Human Papillomavirus (HPV) genotype 18 variants in patients with clinical manifestations of HPV related infections in Bilbao, Spain

**DOI:** 10.1186/1743-422X-9-258

**Published:** 2012-11-02

**Authors:** Sara L Arroyo, Miren Basaras, Elixabete Arrese, Silvia Hernáez, Daniel Andía, Valentín Esteban, Koldo Garcia-Etxebarria, Begoña M Jugo, Ramón Cisterna

**Affiliations:** 1 Immunology, Microbiology and Parasitology Department, University of Basque Country, Leioa, 48940, Spain; 2 Clinical Microbiology and Infection Control Department, Basurto University Hospital, Bilbao, 48013, Spain; 3 Obstetrics and Gynecology Department, Basurto University Hospital, Bilbao, 48013, Spain; 4 Genetics, Physical Anthropology and Animal Physiology Department, University of Basque Country, Leioa, 48940, Spain

**Keywords:** Human papillomavirus infection, Genotype 18, Variants, Recombination, Multiple infection

## Abstract

**Background:**

Human papillomavirus (HPV) variants differ in their biological and chemical properties, and therefore, may present differences in pathogenicity. Most authors classified variants based on the phylogenetic analysis of L1 region. Nevertheless, recombination in HPV samples is becoming a usual finding and thus, characterizing genetic variability in other regions should be essential.

**Objectives:**

We aimed to characterize the genetic variability of HPV 18 in 5 genomic regions: E6, E7, E4, L1 and the Upstream Regulatory Region (URR), working with both single infection and multiple HPV infection samples. Furthermore, we aimed to assess the prevalence of HPV 18 variants in our region and look for possible existence of recombination as well as analyze the relationship between these variants and the type of lesion.

**Methods:**

From 2007 to 2010, Clinical Microbiology and Infection Control Department analyzed 44 samples which were positive for HPV 18. Genetic variability was determined in PCR products and variants were assigned to European, Asian-amerindian or African lineage. Recombination and association of variants with different types of lesion was studied.

**Results:**

Genetic analysis of the regions revealed a total of 56 nucleotide variations. European, African and Asian-amerindian variants were found in 25/44 (56.8%), 10/44 (22.7%) and 5/44 (11.4%) samples, respectively. We detected the presence of recombinant variants in 2/44 (4.5%) cases. Samples taken from high-grade squamous intraepithelial lesions (H-SIL) only presented variants with specific-african substitutions.

**Conclusions:**

Multiple HPV infection, non-european HPV variants prevalence and existence of recombination are considered risk factors for HPV persistence and progression of intraepithelial abnormalities, and therefore, should be taken into consideration in order to help to design and optimize diagnostics protocols as well as improve epidemiologic studies.

Our study is one of the few studies in Spain which analyses the genetic variability of HPV18 and we showed the importance of characterizing more than one genomic region in order to detect recombination and classify HPV variants properly.

## Background

Based on the epidemiologic classification in terms of their risk to induce cervical cancer, human papillomaviruses (HPV) can be divided into 3 groups: “high-risk” genotypes associated with a greater risk of developing cancer, “low-risk” genotypes associated with low grade cell changes or benign epithelium proliferations in the genital area, but not with cancer, and “probable high-risk” genotypes from which there is not enough data about their relationship with cervical cancer to classify them [1].

About 15 genotypes are classified as high-risk types, and two of them (16 and 18) cause over 70% of all cervical cancer cases [2,3]. Nucleotide variability of these genotypes has been largely studied and different molecular variants were described [4,5]. These variants differ in their biological and chemical properties [6-8], and therefore, may become an important risk factor in cervical cancer due to possible differences in pathogenicity.

Most authors classified variants based on the phylogenetic analysis of one genomic region nucleotide variations [9]. Nevertheless, some publications have confirmed the presence of recombination in HPV samples [10,11]. This event may occur due to a homologous recombination or to a repeated infection of the same HPV genotype but different variant and it is more often found since coinfections with more than one HPV type are becoming a usual finding [12-14]. Therefore, it should be essential to determine HPV variants analyzing different genomic regions and multiple infections.

There are very few epidemiological national studies in Spain and all of them refer to HPV 16 which is the most investigated HPV type worldwide. However, there is no previous national work related to HPV 18 nucleotide variability, which is the second most prevalent HPV genotype found in cervical cancer.

The aim of the present study was: i) to characterize the genetic variability of HPV 18 in 5 genomic regions: E6, E7, E4, L1 and the Upstream Regulatory Region (URR), working with both single infection and multiple HPV infection samples, ii) assess the prevalence of HPV 18 variants in our region and look for existence of recombination, and iii) analyze the relationship between variants and types of lesion.

## Results

### Samples collected

Since 2007 to 2010, a total of 1085 positive samples for HPV were received and analyzed. HPV 18 was detected in 65 samples (6%). Forty-four patients consented to have their samples analyzed and studied, so this study was based on their samples: 10 single HPV infections (22.7%) and 34 multiple HPV infections (77.3%).

We were able to amplify HPV DNA in 43/44 samples for E6 region, 41/44 for E7, 35/44 for E4, 43/44 for L1 region and 44/44 for URR region. All PCR products were sequenced and sequences from each region were submitted to GenBank.

### Nucleotide variations

**Figure 1:**
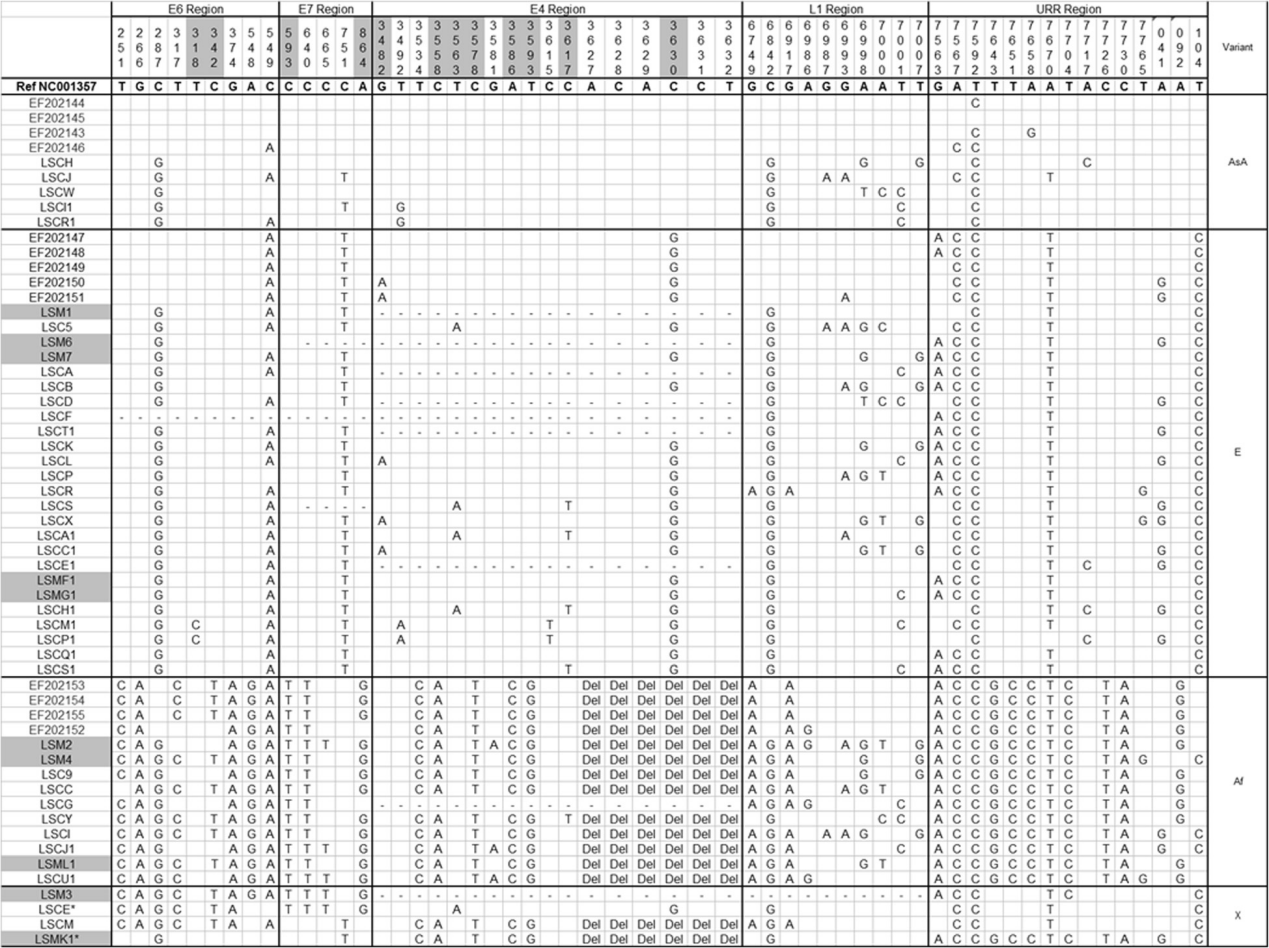
**Nucleotide sequence variations among HPV isolates.** Numbering refers to the first nucleotide of the HPV 18 reference genome (accession number NC001357). Each row indicates the isolate identification and the PCR nucleotide sequence alignment compared to the reference. Isolates EF202143-EF202155 are HPV 18 known variant sequences which belong to Asian-amerindian lineage, African and European lineage. Nucleotide positions where a substitution leads to a change of amino acid are highlighted in gray. In the first column, samples that are highlighted in gray correspond to single HPV infection samples, whereas not highlighted samples correspond to multiple HPV infections. Dashes indicate absence of nucleotide sequence data. Recombinant variants are indicated by an asterisk.

Variant distribution was determined through E6, E7, E4, L1 and URR sequences. Genetic analysis of the regions revealed a total of 56 nucleotide variations (Figure 1).

In the *E6* gene nine nucleotide variations were detected. Six of them were specific to the African lineage: T317C (6/10 African variants), T251C (9/10), A548G, G266A and G374A (present in all African isolates) and C342T (5/10 African variants) which lead to a non-synonymous amino acid alteration His/Tyr. A non-synonymous substitution T318C (Tyr/His) was found to be specific to the European lineage (2/25 European isolates), while the synonymous substitution C549A was detected among the three different branches (35/43 sequenced *E6* amplimers). In our study, C287G was observed in all HPV 18 isolates.

*E7* gene genetic variability analysis revealed five nucleotides substitutions. Three nucleotide variations were specific for the African lineage: C665T (3/10 African variants), C593T (His/Tyr), C640C and T864G (Asn/Ser). All of them but C665T and T864G were present in all African variants. One synonymous substitution (C751T) was detected in both European and Asian-amerindian isolates (26/34 non-African variants).

*E4* gene analysis presented most nucleotide variations (17 substitutions) and almost half of them (8/17) lead to amino acid changes. All African variants showed 4 non-synonymous substitutions (C3558A His/Gln, C3578T Ser/Leu, A3586C Ser/Arg and T3593G Ile/Ser), one synonymous variation (T3534C) and a deletion of 6 amino acids (3627–3632). European variants showed 4 specific non synonymous substitutions G3482A (Ser/Asn, 3/25 isolates), T3563A (Leu/Gln, 4/25), C3617T (Ser/Leu, 4/25) and C3630G (His/Gln, all European isolates). Two non-synonymous substitutions were also detected in two European isolates: T3492A and C3615T.

*L1* gene and URR sequence analysis demonstrated the presence of substitutions C6842G and T7592C in all our isolates.

Most nucleotide variations found in our study have been already described in literature except for T318C, C665T, C3615T, C3617T, G6897A, G6993A, A7000T/C, T7001C, T7007G and T7765G. Only substitutions in positions 318 and 3617 lead to amino acid changes (Tir/His and Ser/Leu, respectively). T318C substitution was present in 2 European isolates whereas C3617T nucleotide change was not specific to any lineage and was detected in 5 samples.

### HPV variants

In our study, the predominant variant found was the European (25/44 samples) followed by the African (10/44) and the Asian-amerindian variants (5/44).

**Figure 2:**
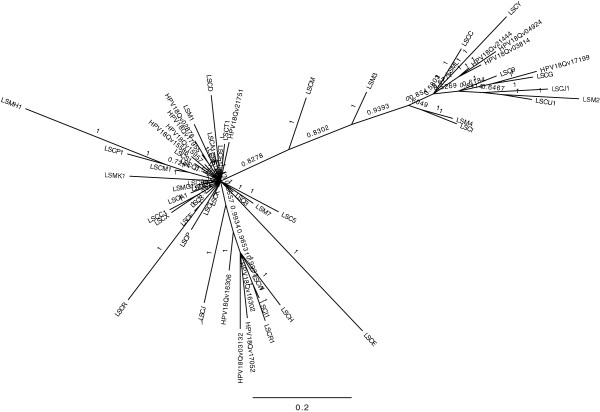
**Phylogenetic tree of the HPV 18 isolates.** E6, E7, E4, L1 and URR nucleotide sequences of isolates using the Bayesian inference method implemented in MrBayes 3.1. Isolates EF202143-EF202155 are included as HPV 18 reference variant sequences which belong to Asian-amerindian lineage, African and European lineage.

Phylogenetic analysis of the all regions studied showed that European and Asian-amerindian lineages formed closely related nodes as well as a maximal nucleotide diversity between African and non-African variants (Figure 2) (Additional file 1).

Isolates which showed nucleotide diversity from the three branches (European, Asian-amerindian and African) - LSM3, LSCM, LSCE, LSMK1, LSCR, LSMH1, LSC5, LSM7 and LSCB – were analyzed for possible recombination (Table 1).

**Table 1:** Evidence for recombinant samples

**Sample \ Gene**	**E6**	**E7**	**E4**	**L1**	**URR**	**Program inference**
LSM3	Af	Af	X	X	E	No recombination detected.
LSCE	Af	Af	E	E	E	Maxchi detected recombination. (LSCM is one of the donors)
LSMK1	AsA	E	Af	E	Af	Maxchi detected recombination. (LSCI and LSCR1 are the donors)
LSCR	E	E	E	E	E	No recombination detected.
LSMH1	E	E	E	E	E	No recombination detected.
LSC5	E	E	E	E	E	No recombination detected.
LSM7	E	E	E	E	E	No recombination detected.
LSCB	AsA	E	E	E	E	No recombination detected.
LSCM	Af	E	Af	Af	E	No recombination detected.

Isolates analyzed for possible recombination. Phylogenetic trees were constructed for each sequenced region and isolates were classified as Asian-amerindian (AsA), European (E) or African (Af) variants. RDP, Maxchi and Chimaera were used for the detection of recombination. URR: Upstream regulatory region, X: absence of data.

Phylogenetic trees were constructed for each sequenced region from these isolates and we found that samples LSCR, LSMH1, LSC5 and LSM7 belonged to the European branch in all regions and therefore, were classified as Europeans. Isolate LSCB belonged to the European lineage in all regions but in *E6* (Asian-amerindian) due to the lack of one nucleotide substitution (C549A). This sample was also classified as European.

Samples LSM3, LSCM, LSCE, LSMK1 belonged to the African branch in some regions but were classified as European in others (Table 1).

RDP [15], Maxchi [16] and Chimaera were used for the detection of recombination in these 9 samples and only 2 of them were found to be recombinant, one single (LSMK1) and one multiple HPV infection (LCE) (Table 1). LSCM and LSM3 were classified as X variants (unknown).

### Variants, type of lesion and infection type

Out of 44 samples, 32 were classified by pathologists as normal (no lesion was found), 9 samples were diagnosed as L-SIL while presence of H-SIL was detected in 3 specimens.

High grade lesions only presented African variants (2/3 isolates, 66.7%) and variants that presented both African and European substitutions (1/3 isolates, 33.3%) whereas most European and Asian-amerindian variants were detected in negative cytologies (Table 2).

**Table 2:** Human papillomavirus 18 variants vs type of lesion

**Variants \ Type of Lesion**	**Neg**	**L-SIL**	**H-SIL**
European	**71.0%**	3.3%	0%
Asian-amerindian	12.9%	1.1%	0%
African	12.9%	4.4%	**100%**
Recombinant	3.2%	1.1%	0%

Neg: no lesion, L-SIL: low-grade squamous intrapehitelial lesion, H-SIL: high-grade squamous intraephitelial lesion.

Presence of lesions associated with non-European variants was found to be statistically significant (p = 0.01053). Nevertheless, there was not a statistically significant association between type of infections (single vs multiple) and presence of lesions (p = 0.18078).

## Discussion

There are almost no epidemiologic studies about HPV 18 variants carried out in Spain and even though national HPV prevalence is low, it cannot be forgotten that this genotype together with genotype 16 cause 70% of cervical cancer cases.

Many authors confirm that distribution of HPV variants is related to geographic or race distribution [6,17] and therefore, Spain should expect predominance of European variant, followed by African and Asian-amerindian variants. Our results show concordance with this stating: 25 European variants (56.8%), 10 African (22.7%) and 5 Asian-amerindian variants (11.4%).

HPV 18 has been associated with both recurrent lesions with very bad clinical prognosis [18] and benign lesions [19]. This fact may reflect the oncogenic potential difference among variants. Hecht et al [20] identified a HPV 18 variant with lower oncogenic potential due to its absence in cervical cancer but presence in 40% of intraepithelial lesions. Villa et al [21] suggested that non-European HPV 18 variants persisted more frequently and were more associated with pre-invasive lesions. Since then, most studies confirm that different variants of the same genotype differ in their pathogenic characteristics and therefore, nucleotide substitutions may play an important role. Our study results show concordance with these statements as African variants and variants where most of specific-african substitutions were detected were the only type of variants detected in H-SIL.

Most nucleotide changes reported in our study have been previously described and some of them are of particular importance. In URR, the mutation A41G is located in the Sp-1 binding site and isolates with this nucleotide variation have shown to have an increased transcriptional activity [22]. Variations in positions 41 and 104 modulate Sp1 and YY1 activities and are associated to a higher activity of the E6/E7 promoter. Patients with T104C substitution are less likely to present tumour recurrence [23]. Other nucleotide changes like T7651C, A7658C and C7726T also lay within transcription factor binding sites.

Substitutions C287G, C6842G and T7592C were found in all our isolates. Variation C6842G has been previously reported as error in the original sequence [24], and H. Arias–Pulido et al. sequenced the original reference HPV 18 plasmid (provided by E-M de Villiers, Deutsches Krebsforschungzentrum, Germany) and observed the substitution T7592C [25], so it is considered as a sequencing error in the original HPV 18 reference sequence report.

Furthermore, ten “new” nucleotide variations have been detected and two of them were non-synonymous and lead to amino acid changes (T318C and C3617T, Tir/His and Ser/Leu, respectively).

Knowledge on HPV variants and their nucleotide variability is essential for three main reasons: i) nucleotide variations may interfere with the viral oncogenic potential, ii) host cellular immune response can be different when there are substitutions in the amino acids on the viral capsid which may be relevant for the vaccination, iii) HPV infections with a variant may not give immunological protection against a subsequent infection with other variant of the same genotype.

Nowadays, HPV variants recombination has already been described and it is more often found since coinfection with more than one HPV type prevalence is not a unusual finding [10].

In our study we detected 2 recombinant variants (4.5%) which might have been missed or wrong classified if only amplifying one genomic region. Furthermore, non-recombinant samples as LSCM or LSM3 showed specific-african substitutions in some regions (for example E6) whereas they would be classified as european variants when only analyzing nucleotide variation in URR. Therefore, characterizing more than one genomic region may be essential in order to detect recombination and classify HPV variants properly.

We amplified URR and *E6*, *E7* and *L1*genes from at least 93% of samples. However, when characterizing E4 region, we were only able to amplify 35 samples. *E4* gene is generally disrupted during DNA integration into the host genome and this disruption may explain the inability to amplify *E4* gene in some of our samples.

In conclusion, data and knowledge on geographic HPV intratypic variants distribution might help to establish a data base about the diversity and pathogenicity of different HPV variants, which may help to design and optimize diagnostics protocols in order to reduce the disease.

## Methods

### Recruitment of participants

Clinical Microbiology and Infection Control Department at Basurto University Hospital (Basque Country, North of Spain) analyzed samples which were remitted from different Hospital Services, especially the Consultation of Sexually Transmitted Diseases and the Department of Obstetrics and Gynecology, from 2007 to 2010.

All samples were collected from patients with clinical manifestations of HPV related infections. Lesions were classified by pathologists into three categories: negative (no lesion was found), low-grade squamous intraepithelial lesion (L-SIL) or high-grade squamous intraepithelial lesion (H-SIL).

Molecular genotyping was carried out using “Linear Array HPV Genotyping Test” kit (Roche Molecular Diagnostics). In our study, we analyzed positive samples for HPV genotype 18 (both single infections and multiple HPV infections) from patients who had given written, informed consent.

### Genomic DNA extraction

DNA extraction was performed by QIAamp DNA mini Kit (Qiagen, Hilden, Germany), according to the manufacturer´s instructions. Extracted DNA was eluted with 200 μl AE buffer and stored at −20°C until amplification.

### PCR amplification and sequencing

Amplification of HPV *E6, E7, E4* genes and the URR was performed using type-specific primers designed according to HPV 18 genome prototype sequence (GenBank accession number NC001357). The URR was amplified using 2 primer sets. In order to amplify L1 region, consensus HPV primers were used (Table 3).

**Table 3:** Polymerase chain reaction characteristics for Human papillomavirus 18

	**Primer sequence (5′ – 3′)**	**Annealing Temp/Cycles**	Nucleotides amplified*	**Amplicon size**
E6 F	AGTAACCGAAAACGGTCGGGA	55°C/40 cycles	38-491	454 pb
E6 R	GTTGTGAAATCGTCGTTTTTCA			
E7 F	TGAAAAACGACGATTTCACAAC	55°C/40 cycles	470-931	462 pb
E7 R	ACCTTCTGGATCAGCCATTG			
E4 F	GTAAAGGAAGGGTACAACACG	57°C/35 cycles	3309-3792	484 pb
E4 R	CTGTCCAATGCCAGGTGGA			
LCR 1 F	TCGGTTGCCTTTGGCTTAT	55°C/40 cycles	7465-7775	311 pb
LCR 1 R	AAGGGTAGACAGAATGTTGGACA	55°C/40 cycles	7718-163	303 pb
LCR 2 F	GCTAATTGCATACTTGGCTTG			
LCR 2 R	TCCGTGCACAGATCAGGTAG			
MY11	GCACAGGGTCATAACAATGG	55°C/40 cycles	6558-7012	455 pb
MY09	CGTCCAAGGGATATTGATC			
L1 seq	ACAGTCTCCTGTACCTGGG			

*Position numbering refers to the first nucleotide of the HPV 18 reference genome (accession number NC001357). F: forward primer, R: reverse primer.

PCR was performed in 30 μl of reaction mixture containing 10 × PCR buffer, 25 mmol/L MgCl_2_, 25 mmol/L of each deoxynucleoside, 100 pmol/L of sense and anti-sense primer, 5 μl of template DNA and 2,5 U of Taq DNA polymerase (Qiagen).

The thermal program started with a pre-heat of 95°C for 15 min, followed by 35–40 cycles of suitable annealing temperature which depended on the primers and finished with a final extension at 72°C for 10 min (Table 3).

PCR products were confirmed based on specific bands of amplified DNA presence in agarose gel (2%). Afterwards, amplimers were automatically sequenced using the “Big Dye Terminator Cycle Sequencing kit” (Applied Biosystems) according to the manufacturer´s instructions.

For E6, E7, E4 and URR amplicons the same forward specific primers as those used in amplification were chosen as sequencing primers. In the L1 region, a specific primer was used in order to sequence HPV 18 and not other HPV types present in cases of multiple infection (Table 3).

### Nucleotide variations, phylogenetic analysis: variants and recombination

HPV sequences were aligned and compared to the HPV 18 prototype sequence which belongs to the Asian-amerindian lineage (accession number NC001357), using BioEdit Sequence Alignment Editor v7.0.4.1 and Clustal W (http://www.genome.jp/tools/clustalw/).

Amplification and sequencing of the samples were repeated to confirm nucleotide variations which were present in less than three isolates.

Sequences were assigned to a lineage on the basis of their similarity to HPV 18 known variant sequences [26] which belong to Asian-amerindian lineage (GenBank accession numbers: EF202143 - EF202146), African (EF202152 - EF202155) and European lineage (EF202147- EF202149, EF202151). Phylogenetic trees were built using the Bayesian inference method implemented in MrBayes 3.1 [27] and three methods (all implemented in RDP3 [15]) were used for the detection of recombination (RDP [15], Maxchi [16] and Chimaera) to analyze isolates which did not adjust to the clusters.

### Lesions

Association of lesions and variants or infection type (single HPV vs multiple HPV infection) was analyzed. Fisher exact test was used for statistically significant association.

### GenBank accession numbers

The following are the GenBank accession numbers for all the sequences used in this analysis. X indicates absence of nucleotide sequence data Table 4.

**Table 4:** GeneBank accession numbers for the sequenced isolates

					
**Isolate**	E6	E7	E4	L1	URR
**LSM1**	JN416211	JN416162	X	JN416262	JN416313
**LSM2**	JN416212	JN416163	JN416121	JN416263	JN416314
**LSM3**	JN416213	JN416164	X	X	JN416315
**LSM4**	JN416214	JN416165	JN416122	JN416264	JN416316
**LSC5**	JN416215	JN416166	JN416123	JN416265	JN416317
**LSM6**	JN416216	X	X	JN416266	JN416318
**LSM7**	JN416217	JN416167	JN416124	JN416267	JN416319
**LSC9**	JN416219	JN416169	JN416125	JN416269	JN416321
**LSCA**	JN416220	JN416170	X	JN416270	JN416322
**LSCB**	JN416221	JN416171	JN416126	JN416271	JN416323
**LSCC**	JN416222	JN416172	JN416127	JN416272	JN416324
**LSCD**	JN416223	JN416173	X	JN416273	JN416325
**LSCE**	JN416224	JN416174	JN416128	JN416274	JN416326
**LSCF**	X	X	X	JN416275	JN416327
**LSCG**	JN416225	JN416175	X	JN416276	JN416328
**LSCH**	JN416226	JN416176	JN416129	JN416277	JN416329
**LSCI**	JN416227	JN416177	JN416130	JN416278	JN416330
**LSCJ**	JN416228	JN416178	JN416131	JN416279	JN416331
**LSCK**	JN416229	JN416179	JN416132	JN416280	JN416332
**LSCL**	JN416230	JN416180	JN416133	JN416281	JN416333
**LSCM**	JN416231	JN416181	JN416134	JN416282	JN416334
**LSCP**	JN416232	JN416182	JN416135	JN416283	JN416335
**LSCR**	JN416234	JN416184	JN416137	JN416285	JN416337
**LSCS**	JN416235	X	JN416138	JN416286	JN416338
**LSCW**	JN416238	JN416187	JN416141	JN416289	JN416341
**LSCX**	JN416239	JN416188	JN416142	JN416290	JN416342
**LSCY**	JN416240	JN416189	JN416143	JN416291	JN416343
**LSCA1**	JN416241	JN416190	JN416144	JN416292	JN416344
**LSCC1**	JN416243	JN416192	JN416146	JN416294	JN416346
**LSCE1**	JN416245	JN416194	X	JN416296	JN416348
**LSMF1**	JN416246	JN416195	JN416147	JN416297	JN416349
**LSMG1**	JN416247	JN416196	JN416148	JN416298	JN416350
**LSCH1**	JN416248	JN416197	JN416149	JN416299	JN416351
**LSCI1**	JN416249	JN416198	JN416150	JN416300	JN416352
**LSCJ1**	JN416250	JN416199	JN416151	JN416301	JN416353
**LSMK1**	JN416251	JN416200	JN416152	JN416302	JN416354
**LSML1**	JN416252	JN416201	JN416153	JN416303	JN416355
**LSCM1**	JN416253	JN416202	JN416154	JN416304	JN416356
**LSCP1**	JN416256	JN416205	JN416157	JN416307	JN416359
**LSCQ1**	JN416257	JN416206	JN416158	JN416308	JN416360
**LSCR1**	JN416258	JN416207	JN416159	JN416309	JN416361
**LSCS1**	JN416259	JN416208	JN416160	JN416310	JN416362
**LSCT1**	JN416260	JN416209	X	JN416311	JN416363
**LSCU1**	JN416261	JN416210	JN416161	JN416312	JN416364

### Ethical approval

All procedures followed were approved by the appropriate Ethics Commitee related to our institutions (Basurto University Hospital and University of Basque Country) and complied with the guidelines and ethical standards for experimental investigation with human subjects of Helsinki Declaration of 1975, as revised in 2000. All study participants provided written, informed consent.

## Abbreviations

HPV: Human papillomavirus; H-SIL: High-grade squamous intraepithelial lesion; L-SIL: Low-grade squamous intraepithelial lesion; SIL: Squamous intraepithelial lesion; URR: Upstream regulatory region.

## Competing interests

All authors declare no potential conflicts of interest.

## Authors’ contributions

LSA carried out the molecular studies, participated in the sequence alignment and drafted the manuscript. MB and EA participated in the design of the study and its coordination and have been involved in drafting the manuscript and revising it critically for important intellectual content. SH, DA and VE have made substantial contributions to acquisition of samples and data and have revised the manuscript critically. KGE and BMJ performed phylogenetic and statistical analysis. RC conceived the study, participated in its design and coordination and has given final approval of the version to be published. All authors read and approved the final manuscript.

## Supplementary Material

Additional file 1 **E4, E6, E7, L1, LCR_bay.** Phylogenetic trees were constructed for each region individually. Click here for file

## References

[B1] Muñoz N Castellsague X de Gonzalez AB Gissmann L HPV in the etiology of human cancer Vaccine 2006 24 1 10 10.1016/j.vaccine.2005.07.047 16949995

[B2] Smith JS Lindsay L Hoots B Keys J Franceschi S Winer R Human papillomavirus type distribution in invasive cervical cancer and high-grade cervical lesions: a meta-analysis update Int J Cancer 2007 121 621 632 10.1002/ijc.22527 17405118

[B3] Muñoz N Bosch FX Castellsagué X Díaz M de Sanjose S Hammouda D Against which human papillomavirus types shall we vaccinate and screen? the international perspective Int J Cancer 2004 111 278 285 10.1002/ijc.20244 15197783

[B4] Yamada T Manos MM Peto J Greer CE Munoz N Bosch FX Human papillomavirus type 16 sequence variation in cervical cancers: a worldwide perspective J Virol 1997 71 2463 2472 9032384 10.1128/jvi.71.3.2463-2472.1997 PMC191357

[B5] Ong CK Chan SY Campo MS Campo MS Fujinaga K Mavromara-Nazos P Evolution of human papillomavirus type 18: an ancient phylogenetic root in Africa and intratype diversity reflect coevolution with human ethnic groups J Virol 1993 67 6424 6431 8411344 10.1128/jvi.67.11.6424-6431.1993 PMC238077

[B6] de Araujo Souza PS Sichero L Macia PC HPV variants and HLA polymorphisms: the role of variability on the risk of cervical cancer Future Oncol 2009 5 359 370 10.2217/fon.09.8 19374542

[B7] Sichero L Ferreira S Trottier H Duarte-Franco E Ferenczy A Franco EL High grade cervical lesions are caused preferentially by non-european variants of HPVs 16 and 18 Int J Cancer 2007 120 1763 1768 10.1002/ijc.22481 17230525

[B8] López-Savedra A González-Maya L de Ponce León S García-Carranca A Mohar A Lizano M Functional implication of sequence variation in the long control region and E2 gene among human papillomavirus type 18 variants Arch Virol 2009 154 747 754 10.1007/s00705-009-0362-4 19337781

[B9] de Villiers EM Fauquet C Broker TR Bernard HU Zur HH Classification of papillomaviruses Virology 2004 324 17 27 10.1016/j.virol.2004.03.033 15183049

[B10] Angulo M Carvajal-Rodríguez A Evidence of recombination within human alpha-papillomavirus Virol J 2007 4 33 10.1186/1743-422X-4-33 17391520 PMC1847806

[B11] Jiang M Xi LF Edelstein ZR Galloway DA Olsem GJ Lin WC Identification of recombinant human papillomavirus type 16 variants Virology 2009 394 8 11 10.1016/j.virol.2009.08.040 19758676 PMC2769496

[B12] Méndez F Muñoz N Posso H Molano M Moreno V van den Brule AJ Cervical coinfection with human papillomavirus (HPV) types and possible implications for the prevention of cervical cancer by HPV vaccines J Infect Dis 2005 192 1158 1165 10.1086/444391 16136457

[B13] Tinelli A Leo G Dell’Edera D Storelli F Galante MM Guido M Molecular methods for a correct diagnosis of multiple HPV infections and clinical implications for vaccine Int J Gynecol Cancer 2011 21 545 550 10.1097/IGC.0b013e31820f5eed 21430458

[B14] Nielson CM Harris RB Flores R Abrahamsen M Papenfuss MR Dunne EF Multiple-type human papillomavirus infection in male anogenital sites: prevalence and associated factors Cancer Epidemiol Biomarkers Prev 2009 18 1077 1083 10.1158/1055-9965.EPI-08-0447 19318438 PMC5415340

[B15] Martin DP Lemey P Lott M Moulton V Posada D Lefeuvre P RDP3: a flexible and fast computer program for analyzing recombination Bioinformatics 2010 26 2462 2463 10.1093/bioinformatics/btq467 20798170 PMC2944210

[B16] Smith J Analyzing the mosaic structure of genes J Mol Evol 1992 34 126 129 1556748 10.1007/BF00182389

[B17] Xi LF Kiviat NB Hildesheim A Galloway DA Wheeler CM Ho J Human papillomavirus type 16 and 18 variants: race-related distribution and persistence J Natl Cancer Inst 2006 98 1045 1052 10.1093/jnci/djj297 16882941

[B18] Im SS Wilczynski SP Burge RA Monk BJ Early stage cervical cancers containing human papillomavirus type 18 DNA have more nodal metastasis and deeper stromal invasion Clin Cancer Res 2003 9 4145 4150 14519638

[B19] Burger R Monk BJ Kurosaki T Anton-Culver H Vasiley SA Berman ML Human papillomavirus type 18: association with poor prognosis in early stage cancer J Natl Cancer Inst 1996 88 1361 1368 10.1093/jnci/88.19.1361 8827013

[B20] Hecht J Kadish A Jiang G Burk R Genetic characterization of the human papillomavirus (HPV) 18 E2 gene in clinical specimens suggests the presence of a subtype with decreased oncogenic potential Int J Cancer 1995 60 369 376 10.1002/ijc.2910600317 7829247

[B21] Villa LL Sichero L Rahal P Caballero O Ferenczy A Rohan T Molecular variants of human papillomavirus types 16 and 18 preferentially associated with cervical neoplasma J Gen Virol 2000 81 2959 2968 11086127 10.1099/0022-1317-81-12-2959

[B22] Rose B Steger G Dong XP Thompson C Cossart Y Tattersall M Point mutations in SP1 motifs in the upstream regulatory region of human papillomavirus type 18 isolates from cervical cancers increase promoter activity J Gen Virol 1998 79 1659 1663 9680128 10.1099/0022-1317-79-7-1659

[B23] Rose B Thompson CH Zhang J Stoeter M Stephen A Pfister H Sequence variation in the upstream regulatory region of HPV 18 isolates from cervical cancers Gynecol Oncol 1997 66 282 289 10.1006/gyno.1997.4740 9264577

[B24] Meissner J Myers G, Baker C, Munger K Sequencing errors in reference HPV clones Human papillomaviruses. A compilation and analysis of nucleic acid and amino acid sequences 1997 3 Los Alamos (NM): Theoretical Biology and Biophysics, Los Alamos National Laboratory 110 123

[B25] Arias-Pulido H Peyton CL Torrez-Martinez N Anderson DN Wheeler CM Human papillomavirus type 18 variant lineages in United States populations characterized by sequence analysis of LCR-E6, E2, and L1 regions Virology 2005 338 22 34 10.1016/j.virol.2005.04.022 15936050

[B26] Chen Z DeSalle R Schiffmn M Herrero R Burk RD Evolutionary dynamics of variant genomes of human papillomavirus types 18, 45 and 97 J Virol 2009 83 1443 1455 10.1128/JVI.02068-08 19036820 PMC2620887

[B27] Ronquist F Huelsenbeck JP MrBayes 3: Bayesian phylogenetic inference under mixed models Bioinformatics 2003 19 1572 1574 10.1093/bioinformatics/btg180 12912839

