# Supplementary figures and images for: Human Papillomavirus (HPV) genotype 18 variants in patients with clinical manifestations of HPV related infections in Bilbao, Spain
Source: Virol J. 2012 Nov 2;9:258. doi: 10.1186/1743-422X-9-258 (PMC3495774; doi:10.1186/1743-422X-9-258)

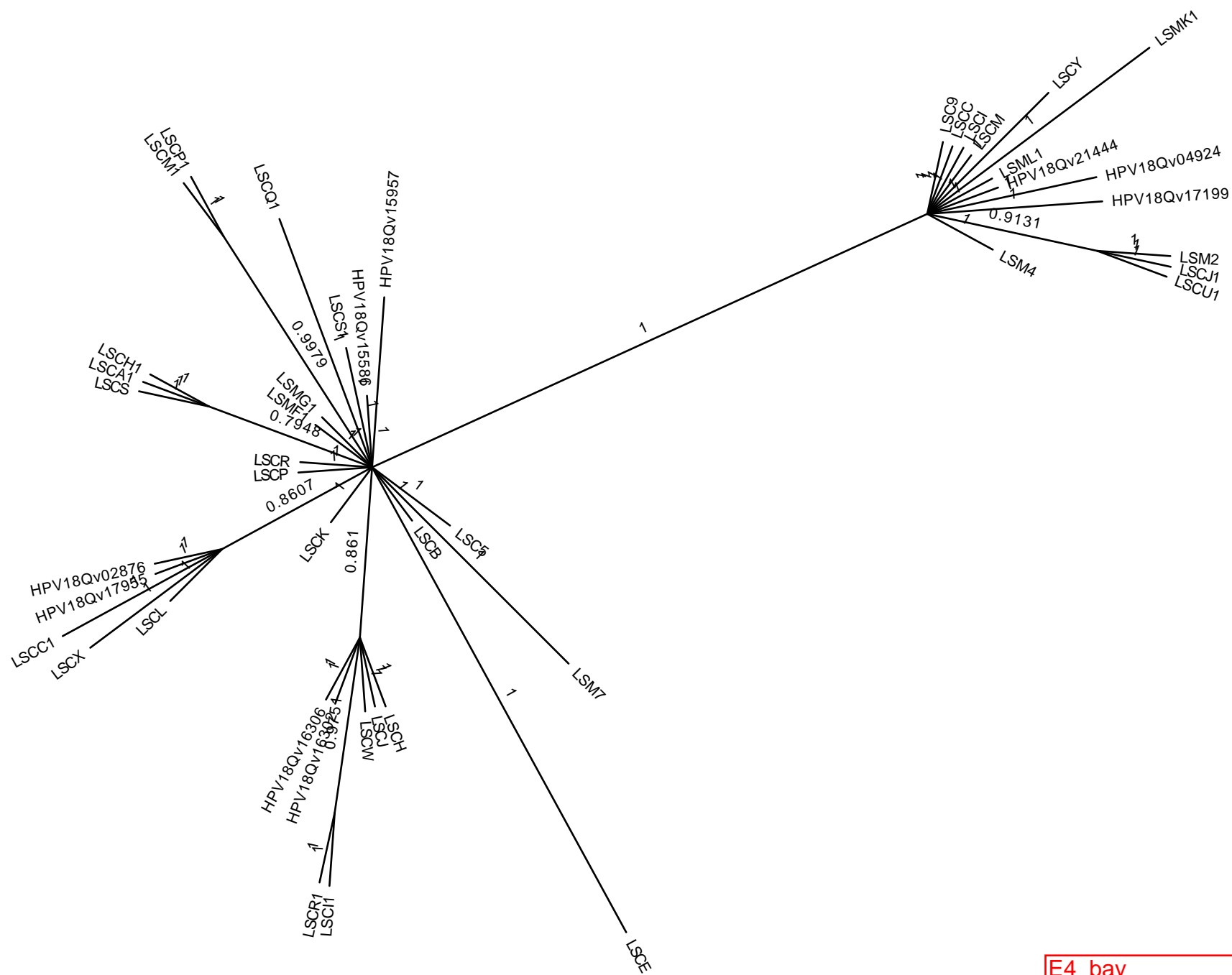

E4\_bay

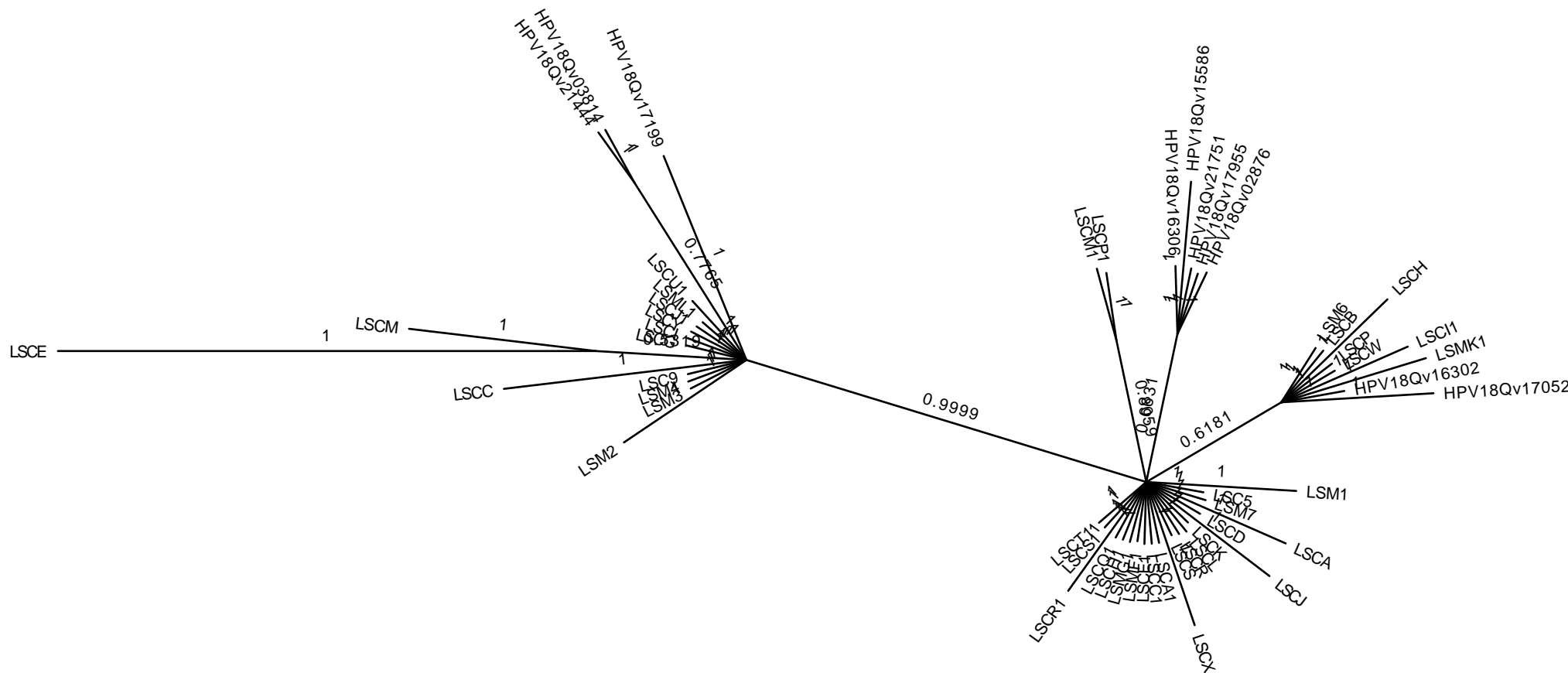

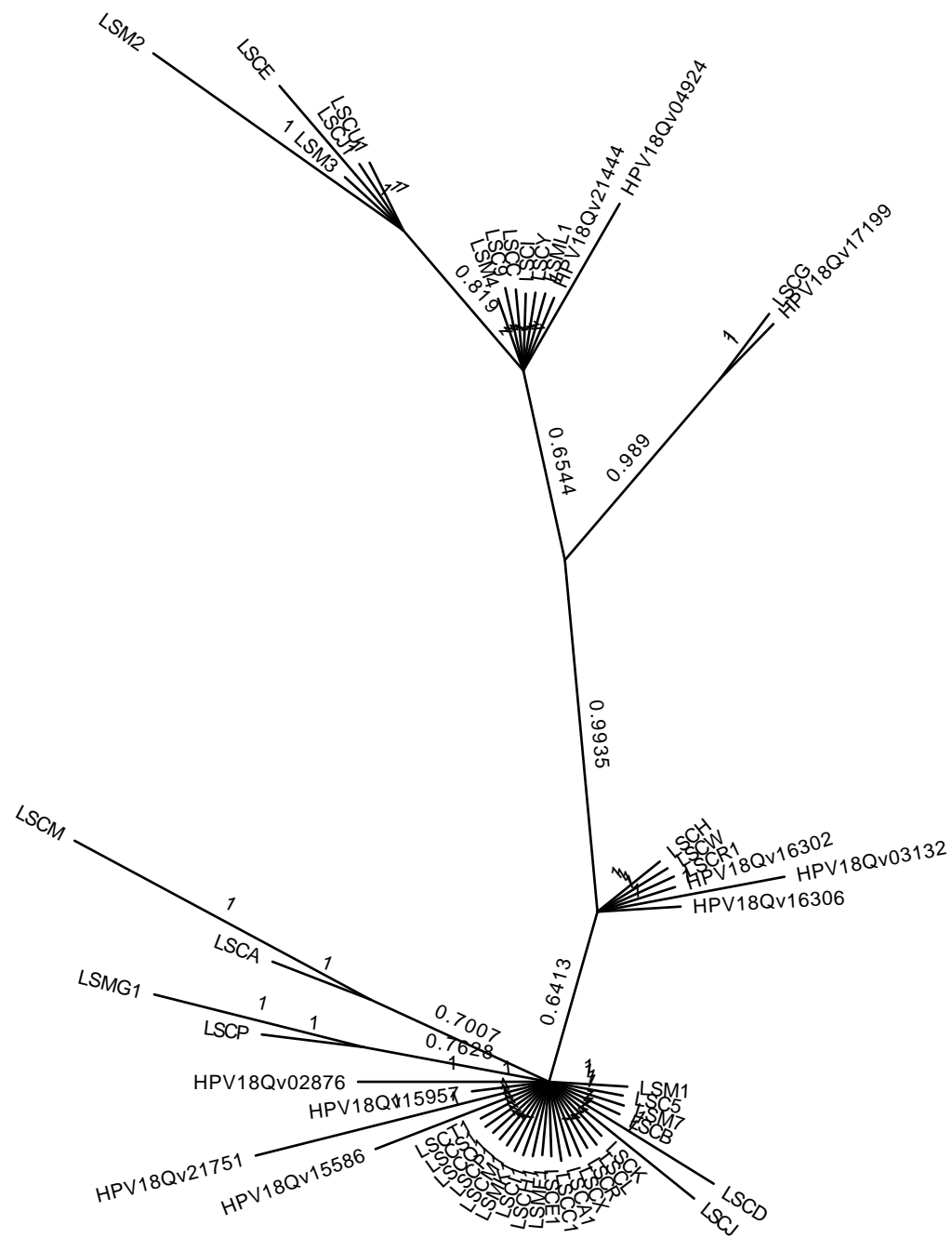

0.09

E7\_bay

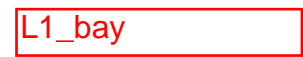

0.06

Supplement: Additional file 1 — E4, E6, E7, L1, LCR_bay. Phylogenetic trees were constructed for each region individually. [file 1743-422X-9-258-S1.pdf]
